# Supplementary material for: Sleep Regularity and Mortality: A Prospective Analysis in the UK Biobank
Source: medRxiv. 2023 Aug 15:2023.04.14.23288550. Originally published 2023 Apr 17. Preprint. [Version 2] doi: 10.1101/2023.04.14.23288550 (PMC10153326; doi:10.1101/2023.04.14.23288550)
Supplement: Supplement 1 [file NIHPP2023.04.14.23288550v2-supplement-1.pdf]

medRxiv preprint doi: https://doi.org/10.1101/2023.04.14.23288550; this version posted August 15, 2023. The copyright holder for this preprint (which was not certified by peer review) is the author/funder, who has granted medRxiv a license to display the preprint in perpetuity.
It is made available under a CC-BY 4.0 International license .

Supplementary appendix

Supplement to: Cribb, Sha, Yiallourou, et al. Sleep Regularity and Mortality: A Prospective Analysis in the UK Biobank

medRxiv preprint doi: https://doi.org/10.1101/2023.04.14.23288550; this version posted August 15, 2023. The copyright holder for this preprint (which was not certified by peer review) is the author/funder, who has granted medRxiv a license to display the preprint in perpetuity.
It is made available under a CC-BY 4.0 International license .

Appendix

Table of Contents

Appendix 1: Removal of low-quality accelerometer data

Appendix 2: STROBE checklist

Table S1: Correlation between sleep regularity index and standard deviation-based regularity metrics

Figure S1. Directed acyclic graph for identification of adjustment variables

Figure S2. Time-varying HRs for 5th and 95th percentiles of SRI (relative to median) for all-cause mortality

Figure S3. Time-varying HRs for 5th and 95th percentiles of SRI (relative to median) for cancer-mortality

Figure S4. SRI and all-cause mortality in sensitivity analyses

Figure S5. SRI and all-cause mortality in sensitivity analyses

Figure S6. SRI and CVD-mortality in sensitivity analyses

Figure S7. SRI and cancer-mortality in sensitivity analyses

medRxiv preprint doi: https://doi.org/10.1101/2023.04.14.23288550; this version posted August 15, 2023. The copyright holder for this preprint (which was not certified by peer review) is the author/funder, who has granted medRxiv a license to display the preprint in perpetuity.
It is made available under a CC-BY 4.0 International license .

Appendix 1: Methods. Removal of low-quality accelerometer data

Accelerometry data of low quality were removed using established UKB criteria; incongruity of self-reported wear time and accelerometer wear time data (5%); insufficient wear time (< 72 hours; 5%); and poorly calibrated data (<1%). Lastly, data were removed for participants in which GGIR was unable to determine a sleep window (5%) and for participants providing less than two valid SRI measurements (i.e., 2 24-hour wear periods; <1%). In total, 88,975 (84%) participants provided valid sleep regularity index data and were included in the study.

medRxiv preprint doi: https://doi.org/10.1101/2023.04.14.23288550; this version posted August 15, 2023. The copyright holder for this preprint (which was not certified by peer review) is the author/funder, who has granted medRxiv a license to display the preprint in perpetuity.
It is made available under a CC-BY 4.0 International license .

Appendix 2: STROBE Statement—Checklist of items that should be included in reports of ***cohort studies***

	Item No	Recommendation
**Title and abstract**	1	(*a*) Indicate the study's design with a commonly used term in the title or the abstract (see title)
		(*b*) Provide in the abstract an informative and balanced summary of what was done and what was found (see pg 2)
**Introduction**		
Background/rationale	2	Explain the scientific background and rationale for the investigation being reported (pg 3)
Objectives	3	State specific objectives, including any prespecified hypotheses (pg 3)
**Methods**		
Study design	4	Present key elements of study design early in the paper (Pg 3)
Setting	5	Describe the setting, locations, and relevant dates, including periods of recruitment, exposure, follow-up, and data collection (Pg 3)
Participants	6	(*a*) Give the eligibility criteria, and the sources and methods of selection of participants. Describe methods of follow-up (Pg 3-4)
		(*b*) For matched studies, give matching criteria and number of exposed and unexposed NA
Variables	7	Clearly define all outcomes, exposures, predictors, potential confounders, and effect modifiers. Give diagnostic criteria, if applicable (Pg 4)
Data sources/ measurement	8*	For each variable of interest, give sources of data and details of methods of assessment (measurement). Describe comparability of assessment methods if there is more than one group (Pg 3-4)
Bias	9	Describe any efforts to address potential sources of bias (pg 5)
Study size	10	Explain how the study size was arrived at (pg 3 and appendix)
Quantitative variables	11	Explain how quantitative variables were handled in the analyses. If applicable, describe which groupings were chosen and why (pg 5)
Statistical methods	12	(*a*) Describe all statistical methods, including those used to control for confounding (pg 4-5, Figure S1)
		(*b*) Describe any methods used to examine subgroups and interactions NA
		(*c*) Explain how missing data were addressed (pg 4-5)
		(*d*) If applicable, explain how loss to follow-up was addressed
		(*e*) Describe any sensitivity analyses (pg 5)
**Results**		
Participants	13*	(a) Report numbers of individuals at each stage of study—eg numbers potentially eligible, examined for eligibility, confirmed eligible, included in the study, completing follow-up, and analysed (Pg 3)
		(b) Give reasons for non-participation at each stage
		(c) Consider use of a flow diagram Not considered necessary but can be created upon request
Descriptive data	14*	(a) Give characteristics of study participants (eg demographic, clinical, social) and information on exposures and potential confounders Table 1
		(b) Indicate number of participants with missing data for each variable of interest<br>Missing data were infrequent, as described in methods
		(c) Summarise follow-up time (eg, average and total amount) (pg 5)
Outcome data	15*	Report numbers of outcome events or summary measures over time (pg 5)
Main results	16	(*a*) Give unadjusted estimates and, if applicable, confounder-adjusted estimates and their precision (eg, 95% confidence interval). Make clear which confounders were adjusted for and why they were included Figures and Appendix figures
		(*b*) Report category boundaries when continuous variables were categorized NA
		(*c*) If relevant, consider translating estimates of relative risk into absolute risk for a meaningful time period Figure 2

medRxiv preprint doi: https://doi.org/10.1101/2023.04.14.23288550; this version posted August 15, 2023. The copyright holder for this preprint (which was not certified by peer review) is the author/funder, who has granted medRxiv a license to display the preprint in perpetuity.
It is made available under a CC-BY 4.0 International license .

Other analyses	17	Report other analyses done—eg analyses of subgroups and interactions, and sensitivity analyses (pg 6)
**Discussion**		
Key results	18	Summarise key results with reference to study objectives (pg 7)
Limitations	19	Discuss limitations of the study, taking into account sources of potential bias or imprecision. Discuss both direction and magnitude of any potential bias (pg 8)
Interpretation	20	Give a cautious overall interpretation of results considering objectives, limitations, multiplicity of analyses, results from similar studies, and other relevant evidence (pg 8)
Generalisability	21	Discuss the generalisability (external validity) of the study results (pg 7-8)
**Other information**		
Funding	22	Give the source of funding and the role of the funders for the present study and, if applicable, for the original study on which the present article is based (pg 8)

*Give information separately for exposed and unexposed groups.

Note: An Explanation and Elaboration article discusses each checklist item and gives methodological background and published examples of transparent reporting. The STROBE checklist is best used in conjunction with this article (freely available on the Web sites of PLoS Medicine at http://www.plosmedicine.org/, Annals of Internal Medicine at http://www.annals.org/, and Epidemiology at http://www.epidem.com/). Information on the STROBE Initiative is available at http://www.strobe-statement.org.

medRxiv preprint doi: https://doi.org/10.1101/2023.04.14.23288550; this version posted August 15, 2023. The copyright holder for this preprint (which was not certified by peer review) is the author/funder, who has granted medRxiv a license to display the preprint in perpetuity.
It is made available under a CC-BY 4.0 International license .

Table S1: Correlation between sleep regularity index and standard deviation-based regularity metrics

Regularity measure	Sleep regularity index	Sleep duration SD	Sleep onset SD
Sleep regularity index	1	-0.32	-0.42
Sleep duration SD	-0.32	1	0.55
Sleep onset SD	-0.42	0.55	1

medRxiv preprint doi: https://doi.org/10.1101/2023.04.14.23288550; this version posted August 15, 2023. The copyright holder for this preprint (which was not certified by peer review) is the author/funder, who has granted medRxiv a license to display the preprint in perpetuity.
It is made available under a CC-BY 4.0 International license .

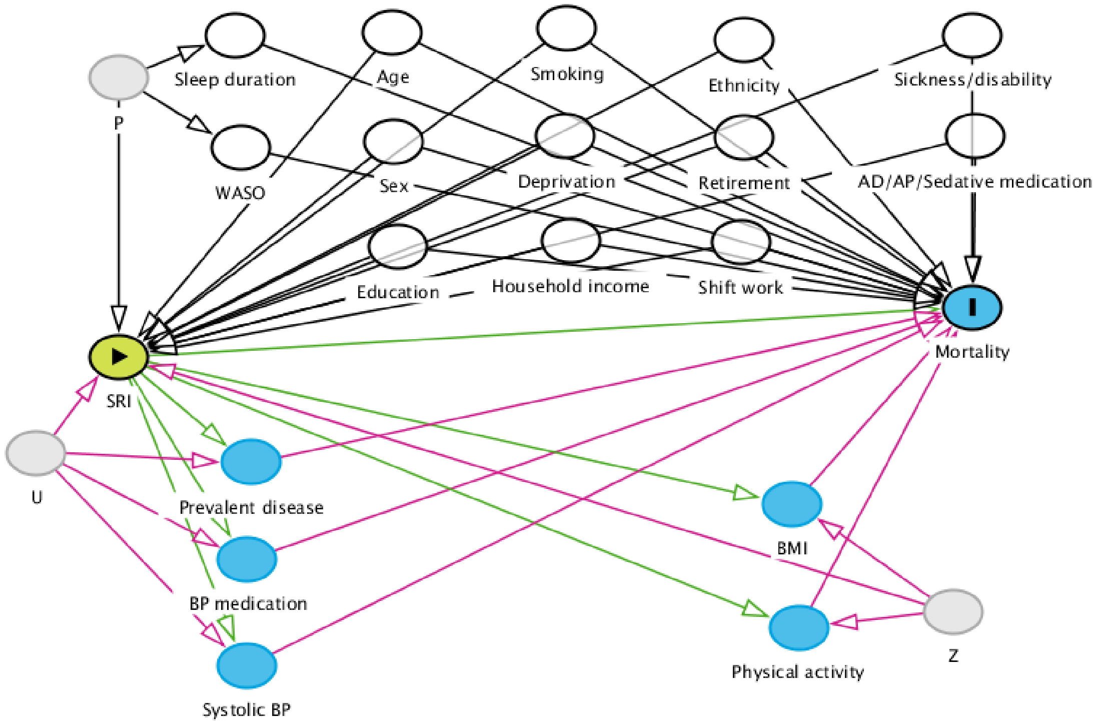

Figure S1. Directed acyclic graph for identification of adjustment variables. The green node indicates the exposure variable (*SRI*), and the blue node (*Mortality*) is the outcome variable. Pale grey nodes indicate unobserved variables; white nodes indicate a variable which has been conditioned on (by regression adjustment or restriction). Paths in red are biasing paths. Arrows indicate the direction of causal effect between two nodes. *P* is an unobserved variable representing unmeasured causes of sleep habits (e.g., genetics). *U* is an unobserved variable representing unmeasured causes of disease and cardiovascular dysfunction (e.g., genetics, biological ageing). *Z* is an unobserved variable representing unmeasured causes of health behaviours (e.g., personality factors, genetics). Green paths from *SRI* to *Prevalent disease*, *BP medication*, *Systolic BP, BMI, and Physical activity* and from these nodes to *Mortality* represent potential mediation of an SRI effect. Conversely, red paths indicate potential sources of confounding (e.g., a backdoor path from *Mortality* to *Prevalent disease* to *SRI* via *U*). Given the current evidence base, we are unable to determine whether and to what extent variables such as *Prevalent disease* act as mediators or confounders (via *U*) of the SRI-mortality association. AP = anti-psychotic; AD = antidepressant; BMI = body mass index; BP = blood pressure; CVD = cardiovascular disease; Deprivation = the Townsend deprivation index; SRI = sleep regularity index; WASO = wake after sleep onset.

medRxiv preprint doi: https://doi.org/10.1101/2023.04.14.23288550; this version posted August 15, 2023. The copyright holder for this preprint (which was not certified by peer review) is the author/funder, who has granted medRxiv a license to display the preprint in perpetuity.
It is made available under a CC-BY 4.0 International license .

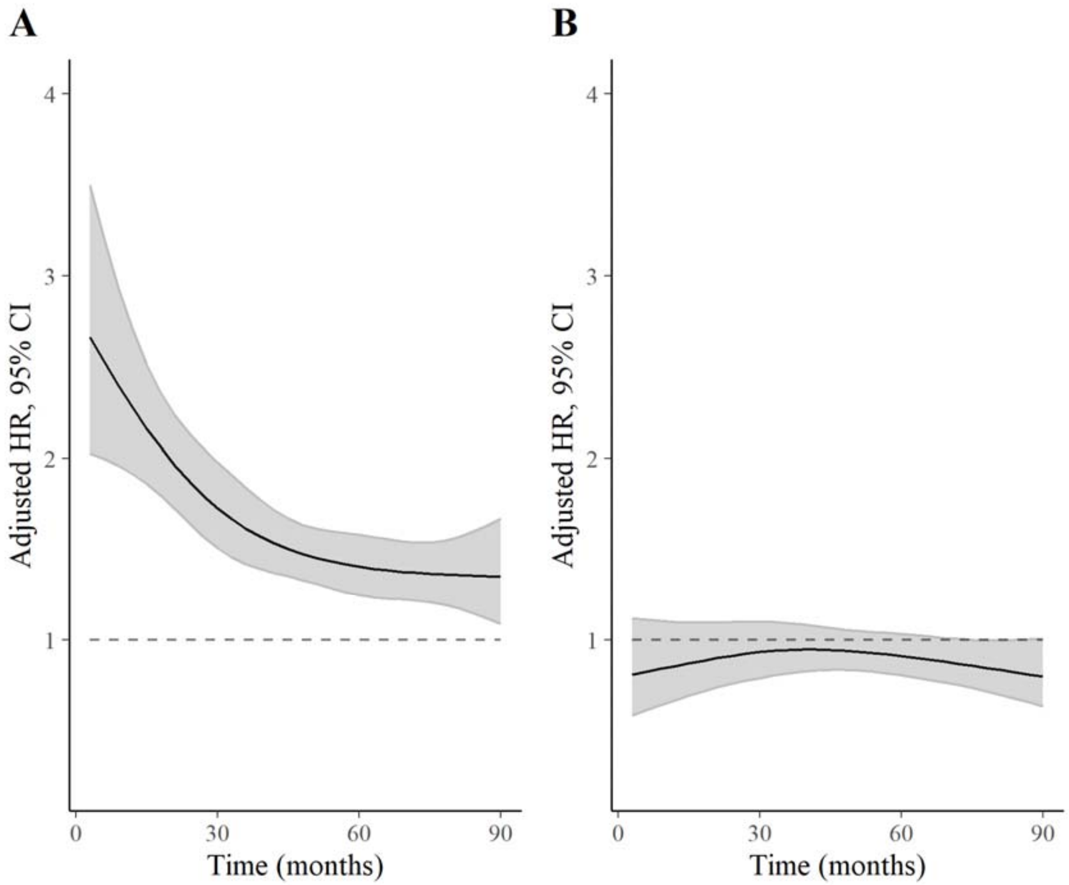

Figure S2. Time-varying HRs for 5th and 95th percentiles of SRI (relative to median) for all-cause mortality. **A:** Hazard ratios for 5th percentile vs median SRI; **B:** Hazard ratios for 95th percentile vs median SRI. Discrete time hazards model including time (aggregated into 3-month intervals and modelled with a restricted cubic spline with knots at the 5th, 35th, 65th, and 95th percentiles), SRI, and an SRI by time interaction. Adjusted for age, Townsend deprivation index, sex, antidepressant, antipsychotic, and sedative medication, ethnicity, household income, education, smoking status (former, current, never), smoking pack years, shift work, retirement status, and sick or disabled (self-reported employment category). All continuous confounders and the SRI were modelled with restricted cubic splines (knots at 10th, 50th, and 90th percentiles) to allow for departures from linearity. There was strong evidence of an interaction between time and SRI (*p* [interaction] < 0.001).

medRxiv preprint doi: https://doi.org/10.1101/2023.04.14.23288550; this version posted August 15, 2023. The copyright holder for this preprint (which was not certified by peer review) is the author/funder, who has granted medRxiv a license to display the preprint in perpetuity.
It is made available under a CC-BY 4.0 International license .

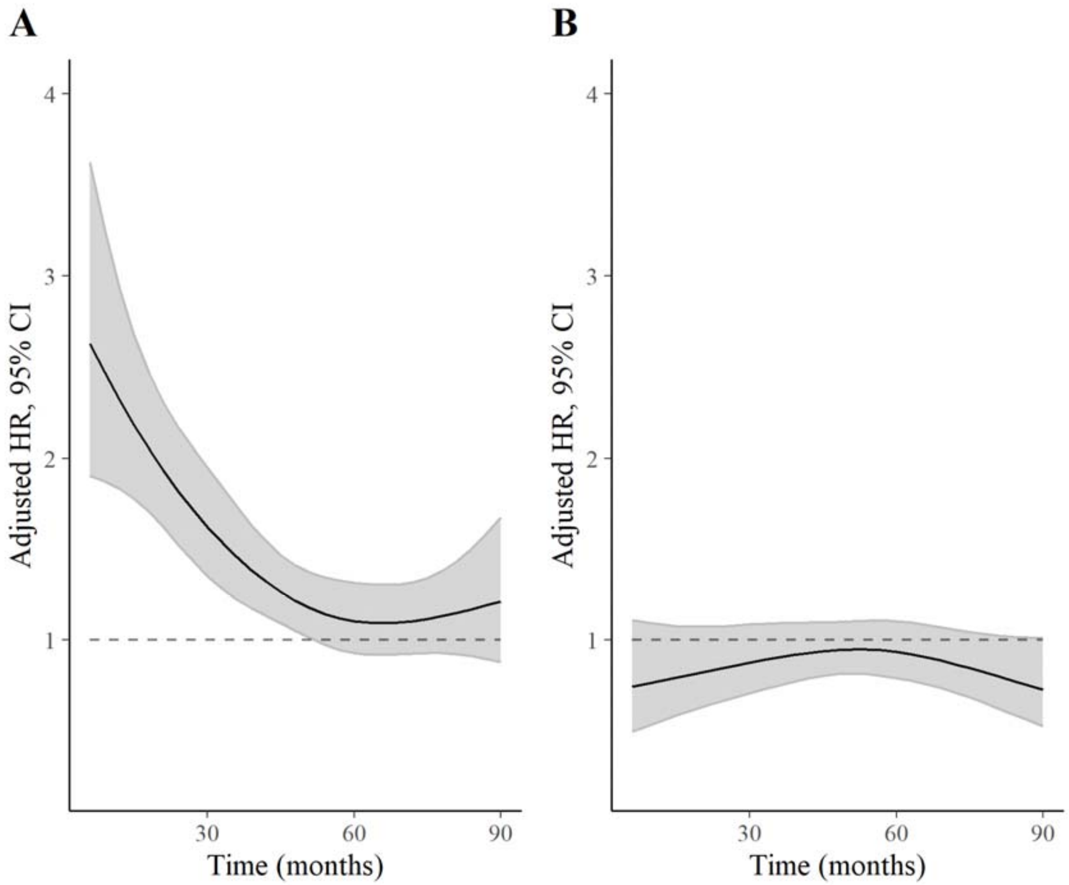

Figure S3. Time-varying HRs for 5th and 95th percentiles of SRI (relative to median) for cancer-mortality. **A:** Hazard ratios for 5th percentile vs median SRI; **B:** Hazard ratios for 95th percentile vs median SRI. Discrete time hazards model including time (aggregated into 3-month intervals and modelled with a restricted cubic spline with knots at the 5th, 35th, 65th, and 95th percentiles), SRI, and an SRI by time interaction. Adjusted for age, Townsend deprivation index, sex, antidepressant, antipsychotic, and sedative medication, ethnicity, household income, education, smoking status (former, current, never), smoking pack years, shift work, retirement status, and sick or disabled (self-reported employment category). All continuous confounders and the SRI were modelled with restricted cubic splines (knots at 10th, 50th, and 90th percentiles) to allow for departures from linearity. There was strong evidence of an interaction between time and SRI (*p* [interaction] < 0.001).

medRxiv preprint doi: https://doi.org/10.1101/2023.04.14.23288550; this version posted August 15, 2023. The copyright holder for this preprint (which was not certified by peer review) is the author/funder, who has granted medRxiv a license to display the preprint in perpetuity.
It is made available under a CC-BY 4.0 International license .

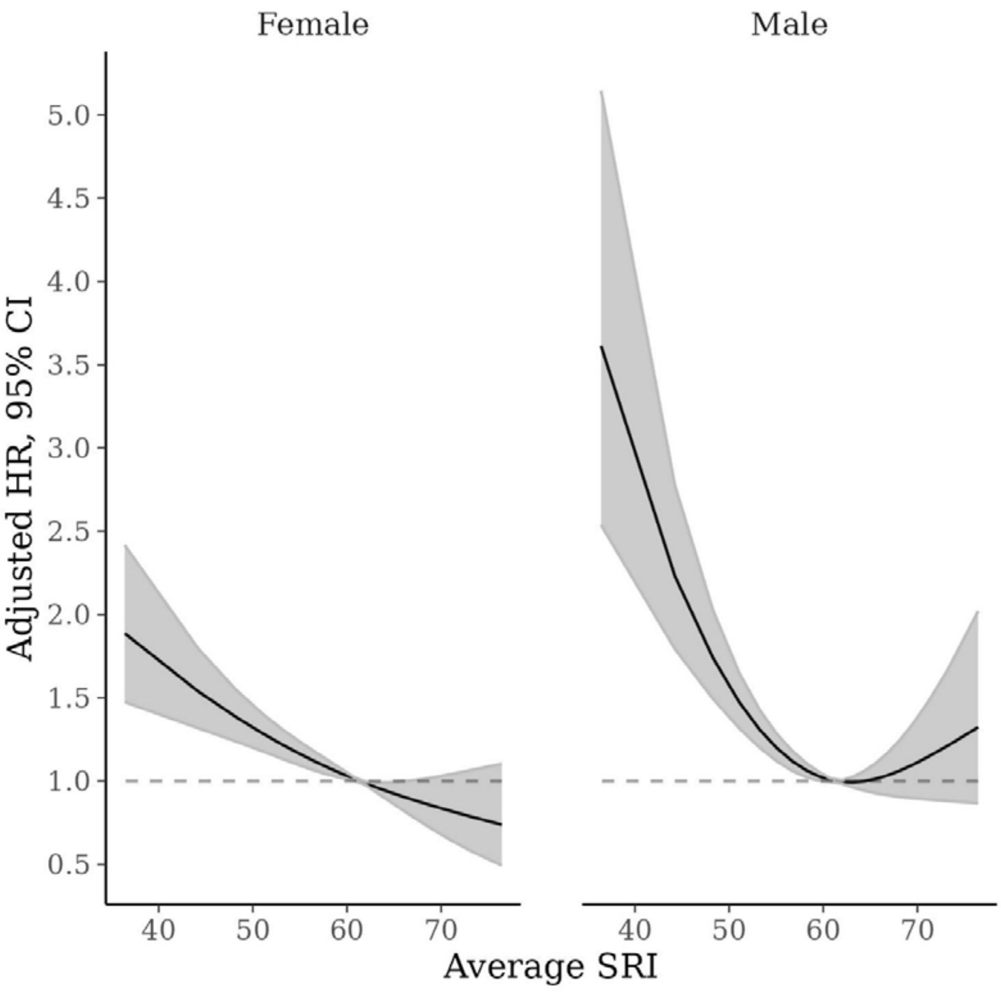

Figure S4. SRI and CVD-specific mortality by sex. Adjusted for age, Townsend deprivation index, antidepressant, antipsychotic, and sedative medication, ethnicity, household income, education, smoking status (former, current, never), smoking pack years, shift work, retirement status, and sick or disabled (self-reported employment category). Hazard ratios are relative to the median SRI (SRI = 60).

medRxiv preprint doi: https://doi.org/10.1101/2023.04.14.23288550; this version posted August 15, 2023. The copyright holder for this preprint (which was not certified by peer review) is the author/funder, who has granted medRxiv a license to display the preprint in perpetuity.
It is made available under a CC-BY 4.0 International license .

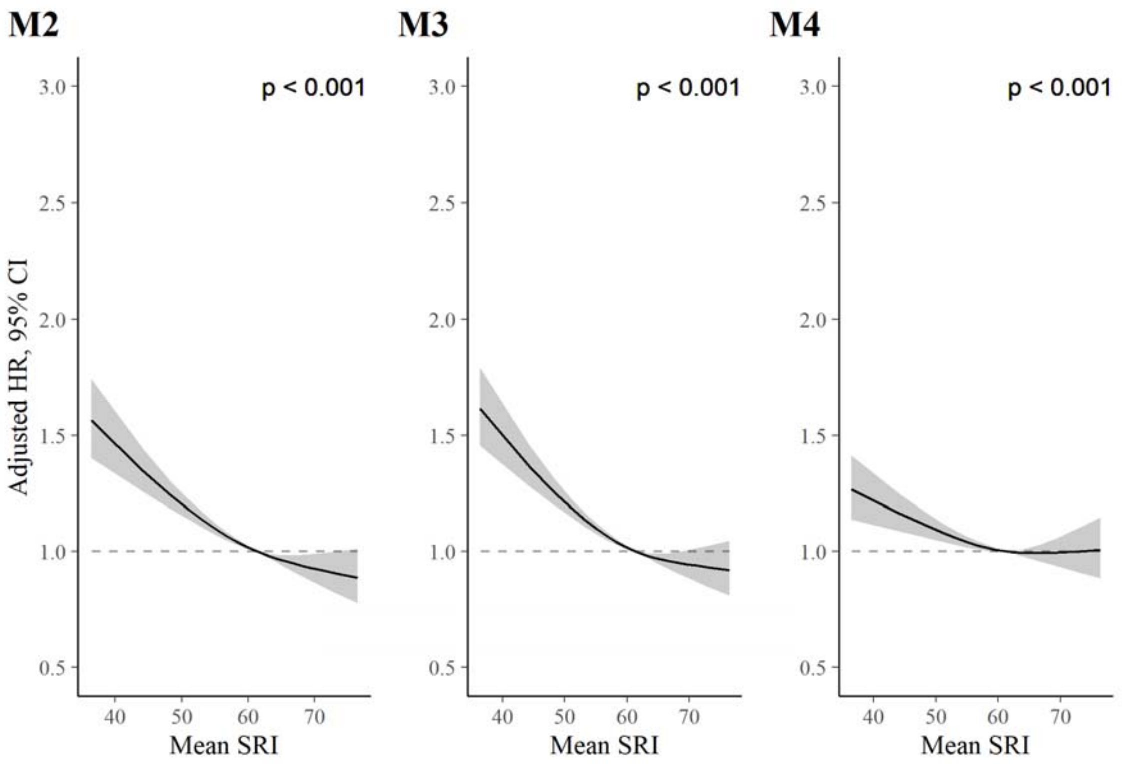

Figure S5. SRI and all-cause mortality in sensitivity analyses. *P* values from global (2 degree of freedom) test of spline term. Hazard ratios are relative to the median SRI (SRI = 60).

Model 2 (M2) adjustments: Adjusted for age, Townsend deprivation index, sex, antidepressant, antipsychotic, and sedative medication, ethnicity, household income, education, smoking status (former, current, never), smoking pack years, shift work, retirement status, and sick or disabled (self-reported employment category), average sleep time, and average wake after sleep onset time. **M2 results:** HRs, relative to the median SRI, were 1.42 (95% CI: 1.31, 1.55) and 0.90 (95% CI: 0.80, 1.00) for SRI at the 5th and 95th percentiles, respectively.

Model 3 (M3) adjustments: Adjusted for age, Townsend deprivation index, sex, antidepressant, antipsychotic, and sedative medication, ethnicity, household income, education, smoking status (former, current, never), smoking pack years, shift work, retirement status, and sick or disabled (self-reported employment category), and past or prevalent diabetes, cancer, mental and behavioural disorder, neurological illness, and cardiovascular illness. **M3 Results:** HRs, relative to the median SRI, were 1.46 (95% CI: 1.35, 1.58) and 0.93 (95% CI: 0.83, 1.03) for the 5th and 95th percentiles of SRI, respectively.

Model 4 (M4) adjustments: Model 3 with additional adjustment for BMI, moderate and vigorous physical activity, systolic blood pressure, and blood pressure medication. **M4 results:** HRs, relative to the median SRI, were 1.20 (95% CI: 1.11, 1.31) and 1.00 (95% CI: 0.90, 1.12) for the 5th and 95th percentiles, respectively.

medRxiv preprint doi: https://doi.org/10.1101/2023.04.14.23288550; this version posted August 15, 2023. The copyright holder for this preprint (which was not certified by peer review) is the author/funder, who has granted medRxiv a license to display the preprint in perpetuity.
It is made available under a CC-BY 4.0 International license .

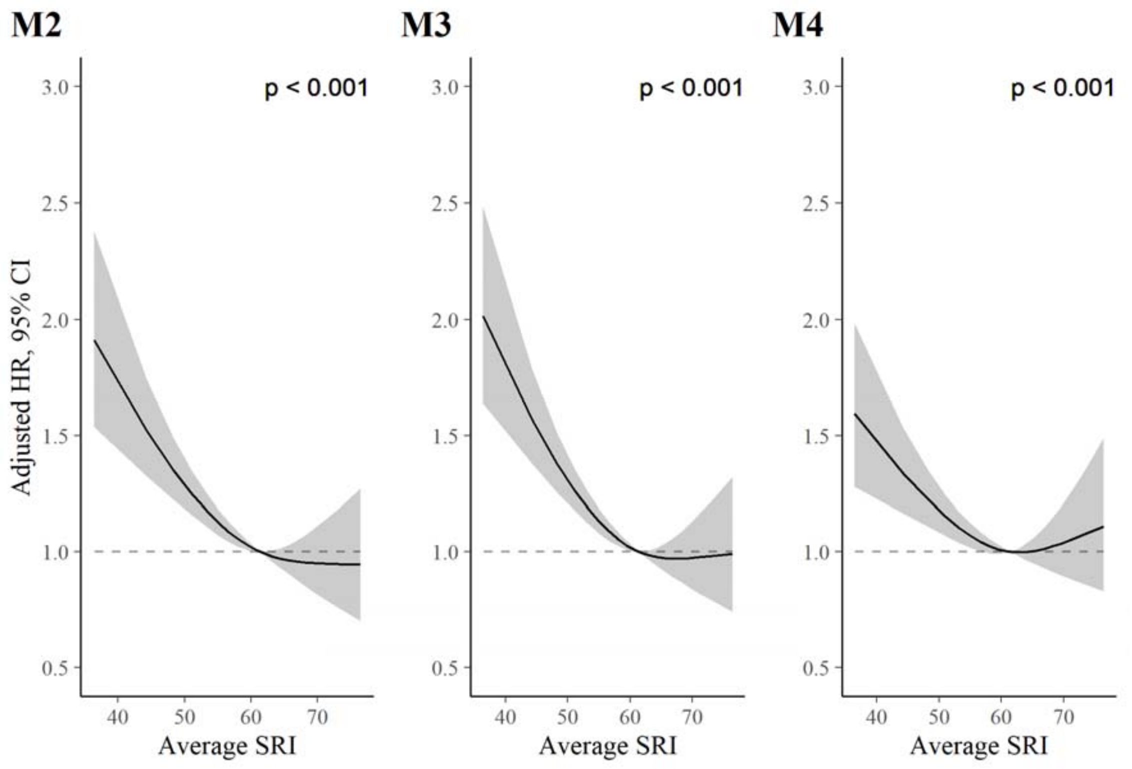

Figure S6. SRI and CVD-mortality in sensitivity analyses. *P* values from global (2 degree of freedom) test of spline term. Hazard ratios are relative to the median SRI (SRI = 60).

Model 2 (M2) adjustments: Adjusted for age, Townsend deprivation index, sex, antidepressant, antipsychotic, and sedative medication, ethnicity, household income, education, smoking status (former, current, never), smoking pack years, shift work, retirement status, and sick or disabled (self-reported employment category), average sleep time, and average wake after sleep onset time. **M2 results:** HRs were 1.66 (95% CI: 1.40, 1.96) and 0.95 (95% CI: 0.73, 1.22) for the 5th and 95th percentile vs. the median SRI, respectively.

Model (M3) adjustments: Adjusted for age, Townsend deprivation index, sex, antidepressant, antipsychotic, and sedative medication, ethnicity, household income, education, smoking status (former, current, never), smoking pack years, shift work, retirement status, and sick or disabled (self-reported employment category), and past or prevalent diabetes, cancer, mental and behavioural disorder, neurological illness, and cardiovascular illness. **M3 results:** HRs were 1.73 (95% CI: 1.47, 2.02) and 0.99 (95% CI: 0.77, 1.26) for the 5th and 95th percentiles, respectively.

Model 4 (M4) adjustments: Model 3 with additional adjustment for BMI, moderate and vigorous physical activity, systolic blood pressure, and blood pressure medication. **M4 results:** HRs were somewhat attenuated: 1.43 (95% CI: 1.21, 1.69) and 1.09 (95% CI: 0.85, 1.40), for the 5th and 95th percentiles, respectively.

medRxiv preprint doi: https://doi.org/10.1101/2023.04.14.23288550; this version posted August 15, 2023. The copyright holder for this preprint (which was not certified by peer review) is the author/funder, who has granted medRxiv a license to display the preprint in perpetuity.
It is made available under a CC-BY 4.0 International license .

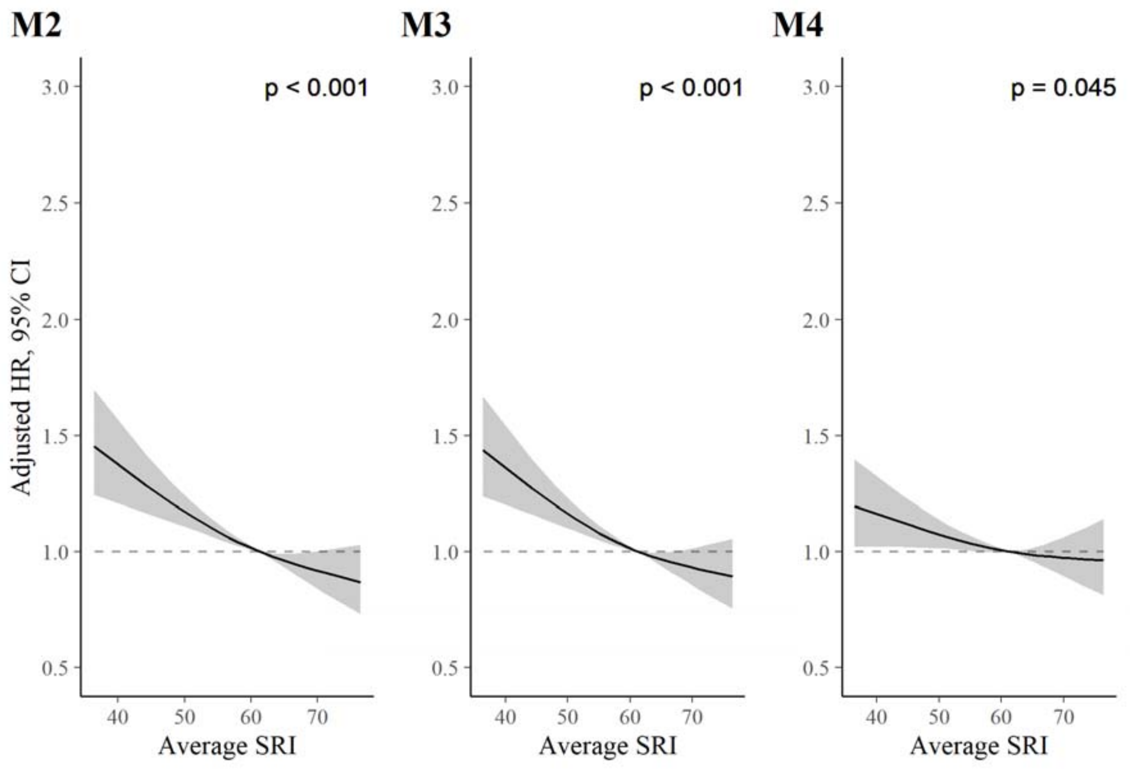

Figure S7. SRI and cancer-mortality in sensitivity analyses. *P* values from global (2 degree of freedom) test of spline term. Hazard ratios are relative to the median SRI (SRI = 60).

Model 2 (M2) adjustments: Adjusted for age, Townsend deprivation index, sex, antidepressant, antipsychotic, and sedative medication, ethnicity, household income, education, smoking status (former, current, never), smoking pack years, shift work, retirement status, and sick or disabled (self-reported employment category), average sleep time, and average wake after sleep onset time. **M2 results:** HRs were 1.35 (95% CI: 1.20, 1.52) and 0.88 (95% CI: 0.76, 1.02) for the 5th and 95th percentile vs. the median SRI, respectively.

Model 3 (M3) adjustments: Adjusted for age, Townsend deprivation index, sex, antidepressant, antipsychotic, and sedative medication, ethnicity, household income, education, smoking status (former, current, never), smoking pack years, shift work, retirement status, and sick or disabled (self-reported employment category), and past or prevalent diabetes, cancer, mental and behavioural disorder, neurological illness, and cardiovascular illness. **M3 results:** HRs were 1.33 (95% CI: 1.19, 1.49) and 0.90 (95% CI: 0.78, 1.04) for the 5th and 95th percentiles, respectively.

Model 4 (M4) adjustments: Model 3 with additional adjustment for BMI, moderate and vigorous physical activity, systolic blood pressure, and blood pressure medication. **M4 results:** HRs were 1.15 (95% CI: 1.02, 1.30) and 0.97 (95% CI: 0.84, 1.12) for the 5th and 95th percentiles, respectively.
